# Supplementary material for: Predictive performance of wearable sensors for mortality risk in older adults: a model development and validation study
Source: medRxiv. 2025 Apr 4:2025.04.03.25325101. Preprint. [Version 1] doi: 10.1101/2025.04.03.25325101 (PMC12191095; doi:10.1101/2025.04.03.25325101)
Supplement: Supplement 1 [file media-1.pdf]

Predictive performance of daily step count and walking cadence for mortality risk in older adults: a model development and validation study

Supplemental Materials

Supplemental Table 1: Power calculations for risk models in the UK Biobank and NHANES 2
Supplemental Table 2: Derivation of risk factors in the UK Biobank 3
Supplemental Table 3: Derivation of risk factors in the NHANES 4
Supplemental Table 4: Codes used to define prior diseases in the UK Biobank 5
Supplemental Table 5: TRIPOD Checklist 6
Supplemental Table 6: Baseline characteristics of the UK Biobank cohort, by sex 7
Supplemental Table 7: Baseline characteristics of the NHANES cohort, by sex 8
Supplemental Table 8: Adjusted hazard ratios for predictors in the UK Biobank, by risk prediction model 9
Supplemental Table 9: Reclassification of participants after addition of accelerometer data to baseline and laboratory models for 5-year predicted mortality risk, by subgroup 10
Supplemental Table 10: Underlying cause of death at five-years in the UK Biobank 11
Supplemental Table 11: Underlying cause of death at five-years in the NHANES 12
Supplemental Table 12: 5-year mortality risk equations 13
Supplemental Table 13: Example 5-year mortality risk score of 65-year-old male 16
Supplemental Figure 1: Flow chart for UK Biobank participants included in analyses 18
Supplemental Figure 2: Flow chart for NHANES participants included in analyses 19
Supplemental Figure 3: Calibration of laboratory models for 5-year all-cause mortality risk in the UK Biobank and the NHANES, by predicted risk decile 20
Supplemental Figure 4: Discrimination improvement after addition of accelerometer data to baseline model for 5-year predicted mortality risk in the UK Biobank, by subgroup 21
Supplemental Figure 5: Discrimination improvement after addition of accelerometer data to laboratory model for 5-year predicted mortality risk in the UK Biobank, by subgroup 22
Supplemental Figure 6: Discrimination improvement after addition of accelerometer data to baseline model for 5-year predicted mortality risk in the NHANES, by subgroup 23
Supplemental Figure 7: Discrimination improvement after addition of accelerometer data to laboratory model for 5-year predicted mortality risk in the NHANES, by subgroup 24
Supplemental Figure 8: Discrimination improvement after addition of accelerometer data to baseline model for 5-year predicted mortality risk, by cause of death 25
Supplemental Figure 9: Discrimination improvement after addition of accelerometer data to laboratory model for 5-year predicted mortality risk, by cause of death 26

Supplemental Table 1: Power calculations for risk models in the UK Biobank and NHANES

Cohort	Model	Number of parameters	Prevalence of 5-year mortality	C-statistic	Estimated number of participants required	Estimated number of events required
UK Biobank	Baseline	20	2.1%	0.75	9,956	210
UK Biobank	Laboratory	40	2.1%	0.75	19,912	419
NHANES	Baseline	20	7.8%	0.80	1,875	147
NHANES	Laboratory	40	7.8%	0.80	3,749	293

Power calculations conducted using the *pmsampsize* R package (version 1.1.3) based on Riley et al. *Statistics in Medicine*. 2019; 38: 1276-1296. NHANES = National Health and Nutrition Examination Survey.

Supplemental Table 2: Derivation of risk factors in the UK Biobank

Risk factor	Units	UKB field ID	Fractional polynomial
Age at accelerometer wear	5-year age groups†	90011; 34; 52	
Sex	Male; Female	31	
Current smoker	Yes; Other	20116	
Body mass index	Kilograms per metre^2	21001	
Systolic blood pressure	Millimetres of mercury	93	
Blood pressure medication	Yes; Other	6177; 6153	
Cholesterol lowering medication	Yes; Other	6177; 6153	
**Prior disease***			
Myocardial infarction	Yes; No	20002	
Stroke	Yes; No	20002	
Congestive heart failure	Yes; No	20002	
Cancer	Yes; No		
Diabetes	Yes; No	20002	
**Laboratory measures**			
Albumin	Grams per litre	30600	
Alanine aminotransferase	Microliter	30620	
Aspartate aminotransferase	Microliter	30650	$AST^2 + log(AST)AST^2$
HDL cholesterol	Millimoles per litre	30760	
Gamma glutamyltransferase	Microliter	30730	
Lymphocyte count	10^9 cells per litre	30120	$Lymphocyte^{-0.5}$
Mean corpuscular haemoglobin	Petagram	30050	
Mean corpuscular haemoglobin concentration	Grams per decilitre	30060	$MCHC^3$
Red blood cell count	10^12 cells per litre	30010	$RBC^3 + log(RBC)RBC^3$
Red blood cell distribution width	Percentage	30070	
Urea	Millimoles per litre	30670	$Urea^{-0.5} + log(Urea)$
White blood cell count	10^9 cells per litre	30000	$WBC^2 + WBC^3$
**Accelerometer data****			
Daily steps	Steps per day		Steps * log (Steps)
Walking cadence	Steps per minute		

†Age groups include <50, 50-54, 55-59, 60-64, 65-69, 70-74, and ≥75 years. *Derived from self-reported and electronic health record data, except cancer where only electronic health records were used. **Measures derived from raw accelerometer data (UKB field ID: 90001). HDL = High density lipoprotein. ID = Identifier. MCHC = Mean corpuscular haemoglobin concentration. RBC = Red blood count. WBC = White blood count.

Supplemental Table 3: Derivation of risk factors in the NHANES

Risk factor	Units	NHANES Field ID	Fractional polynomial
Age at accelerometer wear	5-year age groups†	RIDAGEYR	
Sex	Male; Female	RIAGENDR	
Current smoker	Yes; Other	SMQ040	
Body mass index	Kilograms per metre^2	BMXBMI	
Systolic blood pressure	Millimetres of mercury	BPXSY1-BPXSY3	
Blood pressure medication	Yes; No	BPQ050A	
Cholesterol lowering medication	Yes; No	BPQ100D	
**Prior disease***			
Myocardial infarction	Yes; No	MCQ160E	
Stroke	Yes; No	MCQ160F	
Congestive heart failure	Yes; No	MCQ160B	
Cancer	Yes; No	MCQ220	
Diabetes	Yes; No	DIQ010	
**Laboratory measures**			
Albumin	Grams per litre	LBDSALSI	
Alanine aminotransferase	Microliter	LBXSATSI	
Aspartate aminotransferase	Microliter	LBXSASSI	$AST^2 + log(AST)AST^2$
HDL cholesterol	Millimoles per litre	LBDHDDSI	
Gamma glutamyltransferase	Microliter	LBXSGTSI	
Lymphocyte count	10^9 cells per litre	LBDLYMNO	$Lymphocyte^{-0.5}$
Mean corpuscular haemoglobin	Petagrams	LBXMCHSI	
Mean corpuscular haemoglobin concentration	Grams per decilitre	LBXMC	$MCHC^3$
Red blood cell count	10^12 cells per litre	LBXRBCSI	$RBC^3 + log(RBC)RBC^3$
Red blood cell distribution width	Percentage	LBXRDW	
Urea	Millimoles per litre	LBDSBUSI	$Urea^{-0.5} + log(Urea)$
White blood cell count	10^9 cells per litre	LBXRDW	$WBC^2 + WBC^3$
**Accelerometer data****			
Daily steps	Steps per day		Steps * log(Steps)
Walking cadence	Steps per minute		

†Age groups include <50, 50-54, 55-59, 60-64, 65-69, 70-74, and ≥75 years. *Derived from self-reported data only. **Measures derived from raw accelerometer data (NHANES field ID: PAX80). HDL = High density lipoprotein. ID = Identifier. MCHC = Mean corpuscular haemoglobin concentration. NHANES = National Health and Nutrition Examination Survey. RBC = Red blood count. WBC = White blood count.

Supplemental Table 4: Codes used to define prior diseases in the UK Biobank

Prior disease	ICD-10 diagnosis codes in EHR data	Self-reported conditions at baseline
Myocardial infarction	I21; I22; I25.2	"heart attack/myocardial infarction"
Stroke	I60-I64	"stroke"
Heart failure	I11.0; I13.0; I13.2; I25.5; I42-I43; I50; P29.0	"heart failure/pulmonary oedema"
Cancer	C00-C97 (excluding C44 non-melanoma skin cancer)†	
Diabetes	E10-E14	"diabetes"; "type 1 diabetes"; "type 2 diabetes"

Prior disease in UK Biobank were identified using electronic health record data, where an ICD-10 code was present prior to accelerometer wear (with all participants having a 10-year look back period), or reported by the participant at their baseline assessment (UK Biobank field ID: 20002). †Prior cancer identified in UK Biobank using routinely collected hospital admission and cancer registry data, and excludes non-melanoma skin cancer (ICD-10 code C44). EHR = Electronic Health Record. ICD-10 = International Classification of Diseases, 10th revision.

Supplemental Table 5: TRIPOD Checklist

Section/Topic			Checklist Item	Page
**Title and abstract**				
Title	1	D;V	Identify the study as developing and/or validating a multivariable prediction model, the target population, and the outcome to be predicted.	1
Abstract	2	D;V	Provide a summary of objectives, study design, setting, participants, sample size, predictors, outcome, statistical analysis, results, and conclusions.	2
**Introduction**				
Background and objectives	3a	D;V	Explain the medical context (including whether diagnostic or prognostic) and rationale for developing or validating the multivariable prediction model, including references to existing models.	3
	3b	D;V	Specify the objectives, including whether the study describes the development or validation of the model or both.	
**Methods**				
Source of data	4a	D;V	Describe the study design or source of data (e.g., randomized trial, cohort, or registry data), separately for the development and validation data sets, if applicable.	4
	4b	D;V	Specify the key study dates, including start of accrual; end of accrual; and, if applicable, end of follow-up.	4
Participants	5a	D;V	Specify key elements of the study setting (e.g., primary care, secondary care, general population) including number and location of centres.	4
	5b	D;V	Describe eligibility criteria for participants.	5
	5c	D;V	Give details of treatments received, if relevant.	X
Outcome	6a	D;V	Clearly define the outcome that is predicted by the prediction model, including how and when assessed.	5
	6b	D;V	Report any actions to blind assessment of the outcome to be predicted.	X
Predictors	7a	D;V	Clearly define all predictors used in developing or validating the multivariable prediction model, including how and when they were measured.	4-6
	7b	D;V	Report any actions to blind assessment of predictors for the outcome and other predictors.	X
Sample size	8	D;V	Explain how the study size was arrived at.	7
Missing data	9	D;V	Describe how missing data were handled (e.g., complete-case analysis, single imputation, multiple imputation) with details of any imputation method.	5
Statistical analysis methods	10a	D	Describe how predictors were handled in the analyses.	6
	10b	D	Specify type of model, all model-building procedures (including any predictor selection), and method for internal validation.	5-6
	10c	V	For validation, describe how the predictions were calculated.	6
	10d	D;V	Specify all measures used to assess model performance and, if relevant, to compare multiple models.	6-7
	10e	V	Describe any model updating (e.g., recalibration) arising from the validation, if done.	6
Risk groups	11	D;V	Provide details on how risk groups were created, if done.	6
Development vs. validation	12	V	For validation, identify any differences from the development data in setting, eligibility criteria, outcome, and predictors.	6
**Results**				
Participants	13a	D;V	Describe the flow of participants through the study, including the number of participants with and without the outcome and, if applicable, a summary of the follow-up time. A diagram may be helpful.	7-8
	13b	D;V	Describe the characteristics of the participants (basic demographics, clinical features, available predictors), including the number of participants with missing data for predictors and outcome.	7
	13c	V	For validation, show a comparison with the development data of the distribution of important variables (demographics, predictors and outcome).	7-8
Model development	14a	D	Specify the number of participants and outcome events in each analysis.	7
	14b	D	If done, report the unadjusted association between each candidate predictor and outcome.	X
Model specification	15a	D	Present the full prediction model to allow predictions for individuals (i.e., all regression coefficients, and model intercept or baseline survival at a given time point).	8
	15b	D	Explain how to the use the prediction model.	
Model performance	16	D;V	Report performance measures (with CIs) for the prediction model.	8
Model-updating	17	V	If done, report the results from any model updating (i.e., model specification, model performance).	8
**Discussion**				
Limitations	18	D;V	Discuss any limitations of the study (such as nonrepresentative sample, few events per predictor, missing data).	10-11
Interpretation	19a	V	For validation, discuss the results with reference to performance in the development data, and any other validation data.	10-11
	19b	D;V	Give an overall interpretation of the results, considering objectives, limitations, results from similar studies, and other relevant evidence.	11
Implications	20	D;V	Discuss the potential clinical use of the model and implications for future research.	11
**Other information**				
Supplementary information	21	D;V	Provide information about the availability of supplementary resources, such as study protocol, Web calculator, and data sets.	13
Funding	22	D;V	Give the source of funding and the role of the funders for the present study.	13

Supplemental Table 6: Baseline characteristics of the UK Biobank cohort, by sex

	**Overall (N=79,717)**	**Female (N=44,410)**	**Male (N=35,307)**
Age at accelerometer wear (years)			
<50 years	4,713 (5.9%)	2,741 (6.2%)	1,972 (5.6%)
50-54 years	10,059 (12.6%)	5,983 (13.5%)	4,076 (11.5%)
55-59 years	11,843 (14.9%)	7,123 (16.0%)	4,720 (13.4%)
60-64 years	14,868 (18.7%)	8,711 (19.6%)	6,157 (17.4%)
65-69 years	20,020 (25.1%)	10,879 (24.5%)	9,141 (25.9%)
70-74 years	14,721 (18.5%)	7,346 (16.5%)	7,375 (20.9%)
≥75 years	3,493 (4.4%)	1,627 (3.7%)	1,866 (5.3%)
Current smoker	5,532 (6.9%)	2,603 (5.9%)	2,929 (8.3%)
Body mass index (kg/m^2)	26.7 (4.5)	26.2 (4.8)	27.3 (4.0)
Systolic blood pressure (mmHg)	137 (18.1)	134 (18.5)	140 (16.9)
Blood pressure medication	13,689 (17.2%)	6,036 (13.6%)	7,653 (21.7%)
Cholesterol lowering medication	11,343 (14.2%)	4,126 (9.3%)	7,217 (20.4%)
**Prior disease**			
Myocardial infarction	1,770 (2.2%)	360 (0.8%)	1,410 (4.0%)
Stroke	997 (1.3%)	392 (0.9%)	605 (1.7%)
Congestive heart failure	622 (0.8%)	162 (0.4%)	460 (1.3%)
Cancer	5,583 (7.0%)	3,068 (6.9%)	2,515 (7.1%)
Diabetes	3,276 (4.1%)	1,223 (2.8%)	2,053 (5.8%)
Any of the above prior diseases	10,856 (13.6%)	4,827 (10.9%)	6,029 (17.1%)
**Laboratory data**			
Albumin (g/L)	45.4 (2.6)	45.1 (2.6)	45.7 (2.6)
Alanine aminotransferase (U/L)	22.7 (13.3)	19.6 (11.4)	26.6 (14.4)
Aspartate aminotransferase (U/L)	25.8 (9.3)	24.2 (8.6)	27.8 (9.8)
HDL cholesterol (mmol/L)	1.5 (0.4)	1.6 (0.4)	1.3 (0.3)
Gamma glutamyltransferase (U/L)	34.2 (35.4)	27.9 (29.1)	42.0 (40.7)
Lymphocyte count (10^9 cells/L)	1.9 (0.9)	2.0 (1.0)	1.9 (0.9)
Mean corpuscular haemoglobin (pg)	31.5 (1.8)	31.3 (1.9)	31.7 (1.7)
Mean corpuscular haemoglobin concentration (g/dL)	34.5 (1.1)	34.4 (1.1)	34.6 (1.0)
Red blood cell count (10^12 cells/L)	4.5 (0.4)	4.3 (0.3)	4.8 (0.4)
Red blood cell distribution width (%)	13.4 (0.9)	13.5 (1.0)	13.4 (0.8)
Urea (mmol/L)	5.3 (1.3)	5.1 (1.3)	5.6 (1.3)
White blood cell count (10^9 cells/L)	6.7 (1.8)	6.7 (1.9)	6.7 (1.8)
**Accelerometer data**			
Daily steps (steps per day)	9,030 [6,790-11,700]	8,970 [6,770-11,500]	9,110 [6,830-11,800]
Walking cadence (steps per minute)	114 [106-121]	116 [109-123]	111 [104-117]

Data are number of participants (%), mean (standard deviation) and median [interquartile range]. HDL = High-density lipoprotein.

Supplemental Table 7: Baseline characteristics of the NHANES cohort, by sex

	**Overall (N=4,713)**	**Female (N=2,432)**	**Male (N=2,281)**
Age at accelerometer wear (years)			
<50 years	1,195 (25.4%)	633 (26.0%)	562 (24.6%)
50-54 years	612 (13.0%)	330 (13.6%)	282 (12.4%)
55-59 years	543 (11.5%)	281 (11.6%)	262 (11.5%)
60-64 years	715 (15.2%)	357 (14.7%)	358 (15.7%)
65-69 years	528 (11.2%)	267 (11.0%)	261 (11.4%)
70-74 years	443 (9.4%)	236 (9.7%)	207 (9.1%)
≥75 years	677 (14.4%)	328 (13.5%)	349 (15.3%)
Current smoker	839 (17.8%)	361 (14.8%)	478 (21.0%)
Body mass index (kg/m^2)	29.5 (6.8)	30.0 (7.5)	29.0 (5.9)
Systolic blood pressure (mmHg)	128 (18.7)	127 (19.7)	128 (17.5)
Blood pressure medication	1,895 (40.2%)	1001 (41.2%)	894 (39.2%)
Cholesterol lowering medication	1,437 (30.5%)	711 (29.2%)	726 (31.8%)
**Prior disease**			
Myocardial infarction	264 (5.6%)	96 (3.9%)	168 (7.4%)
Stroke	237 (5.0%)	132 (5.4%)	105 (4.6%)
Congestive heart failure	221 (4.7%)	106 (4.4%)	115 (5.0%)
Cancer	480 (10.2%)	265 (10.9%)	215 (9.4%)
Diabetes	860 (18.2%)	412 (16.9%)	448 (19.6%)
Any of the above prior diseases	1,528 (32.4%)	751 (30.9%)	777 (34.1%)
**Laboratory data**			
Albumin (g/L)	42.2 (3.1)	41.6 (3.0)	42.7 (3.0)
Alanine aminotransferase (U/L)	24.3 (18.2)	21.5 (12.9)	27.2 (22.2)
Aspartate aminotransferase (U/L)	25.7 (14.8)	24.3 (12.8)	27.2 (16.6)
HDL cholesterol (mmol/L)	1.4 (0.4)	1.5 (0.4)	1.2 (0.4)
Gamma glutamyltransferase (U/L)	29.5 (38.1)	26.4 (32.9)	32.8 (42.8)
Lymphocyte count (10^9 cells/Litre)	2.1 (1.2)	2.1 (1.2)	2.0 (1.2)
Mean corpuscular haemoglobin (pg)	30.5 (2.5)	30.2 (2.6)	30.8 (2.3)
Mean corpuscular haemoglobin concentration (g/dL)	33.9 (1.1)	33.8 (1.1)	34.0 (1.0)
Red blood cell count (10^12 cells/L)	4.6 (0.5)	4.4 (0.4)	4.8 (0.5)
Red blood cell distribution width (%)	13.5 (1.4)	13.6 (1.5)	13.4 (1.2)
Urea (mmol/L)	5.1 (2.3)	4.9 (2.2)	5.3 (2.4)
White blood cell count (10^9 cells/L)	7.0 (2.3)	7.0 (2.3)	7.0 (2.3)
**Accelerometer data**			
Daily steps (steps per day)	7,780 [5,120-11,000]	7,270 [4,770-10,200]	8,460 [5,520-12,000]
Walking cadence (steps per min)	103 [95-111]	104 [94-112]	103 [95-110]

Data are number of participants (%), mean (standard deviation) and median [interquartile range]. HDL = High-density lipoprotein. NHANES = National Health and Nutrition Examination Survey.

Supplemental Table 8: Adjusted hazard ratios for predictors in the UK Biobank, by risk prediction model

	Baseline model	Baseline model plus accelerometer data	Laboratory model	Laboratory model plus accelerometer data
Male	1.49 (1.34-1.65)	1.53 (1.38-1.69)	1.43 (1.26-1.64)	1.49 (1.30-1.70)
Age group				
<50 years	Reference	Reference	Reference	Reference
50-54 years	1.53 (0.87-2.67)	1.52 (0.87-2.66)	1.51 (0.87-2.64)	1.51 (0.86-2.64)
55-59 years	2.38 (1.40-4.02)	2.36 (1.39-3.99)	2.31 (1.36-3.91)	2.28 (1.35-3.86)
60-64 years	3.53 (2.12-5.87)	3.44 (2.07-5.73)	3.46 (2.08-5.77)	3.34 (2.00-5.57)
65-69 years	5.02 (3.04-8.29)	4.81 (2.92-7.95)	4.88 (2.95-8.07)	4.61 (2.79-7.63)
70-74 years	7.97 (4.83-13.16)	7.28 (4.41-12.03)	7.39 (4.46-12.24)	6.70 (4.04-11.10)
≥75 years	11.11 (6.64-18.57)	9.48 (5.66-15.88)	9.83 (5.85-16.51)	8.41 (5.00-14.15)
Current smoker	2.27 (1.97-2.63)	2.00 (1.72-2.31)	1.89 (1.62-2.20)	1.71 (1.46-1.99)
Body mass index (per 5 kg/m^2)	1.16 (1.10-1.23)	1.05 (0.99-1.11)	1.13 (1.06-1.20)	1.04 (0.98-1.10)
Systolic blood pressure (per 10 mmHg)	1.08 (1.05-1.10)	1.08 (1.05-1.11)	1.09 (1.06-1.12)	1.09 (1.06-1.12)
Blood pressure medication	1.15 (1.01-1.30)	1.10 (0.97-1.25)	1.07 (0.95-1.22)	1.04 (0.92-1.18)
Cholesterol lowering medication	1.04 (0.91-1.19)	1.04 (0.91-1.19)	1.05 (0.92-1.20)	1.05 (0.91-1.20)
**Prior disease**				
Myocardial infarction	1.51 (1.23-1.86)	1.45 (1.18-1.77)	1.42 (1.16-1.74)	1.38 (1.12-1.69)
Stroke	1.32 (0.99-1.76)	1.16 (0.87-1.54)	1.21 (0.91-1.61)	1.09 (0.82-1.46)
Congestive heart failure	2.34 (1.80-3.03)	1.99 (1.54-2.59)	2.09 (1.61-2.71)	1.85 (1.42-2.40)
Cancer	3.07 (2.73-3.44)	2.95 (2.62-3.31)	2.91 (2.59-3.27)	2.83 (2.51-3.18)
Diabetes	1.52 (1.28-1.80)	1.38 (1.16-1.64)	1.44 (1.21-1.72)	1.34 (1.13-1.60)
**Laboratory measures**				
Albumin (per 10 g/L)			0.65 (0.53-0.79)	0.67 (0.55-0.81)
ALT (per 10 U/L)			0.92 (0.86-0.97)	0.92 (0.87-0.98)
AST (per 10 U/L) FP1			1.07 (1.03-1.10)	1.07 (1.04-1.10)
AST (per 10 U/L) FP2			0.98 (0.97-0.99)	0.98 (0.97-0.99)
HDL cholesterol (per mmol/L)			0.88 (0.75-1.03)	0.93 (0.79-1.08)
Gamma glutamyltransferase (per 100 U/L)			1.14 (1.04-1.25)	1.10 (1.01-1.20)
Lymphocyte count (per 10^9 cells/Litre)			1.60 (1.06-2.39)	1.47 (0.98-2.21)
Mean corpuscular haemoglobin (per 10 pg)			2.34 (1.61-3.40)	2.21 (1.52-3.21)
Mean corpuscular haemoglobin concentration (per 10 g/dL)			0.97 (0.96-0.99)	0.97 (0.96-0.99)
Red blood count (per 10^12 cells/L) FP1			0.96 (0.93-0.99)	0.96 (0.93-0.99)
Red blood count (per 10^12 cells/L) FP2			1.02 (1.01-1.04)	1.02 (1.01-1.04)
Red blood distribution width (per 10%)			3.78 (2.32-6.16)	3.29 (1.99-5.45)
Urea (per 100 mmol/L) FP1			4.48 (2.48-8.09)	3.47 (1.89-6.37)
Urea (per 100 mmol/L) FP2			23.06 (6.53-81.43)	13.91 (3.81-50.78)
White blood count (per 10^10 cells/L) FP1			2.48 (1.75-3.52)	2.11 (1.50-2.97)
White blood count (per 10^10 cells/L) FP2			0.77 (0.66-0.90)	0.81 (0.70-0.94)
**Accelerometer data**				
Daily steps (per 1000 steps) FP1		0.64 (0.57-0.71)		0.68 (0.60-0.76)
Daily steps (per 1000 steps) FP2		1.13 (1.09-1.17)		1.11 (1.07-1.15)
Walking cadence (per 10 steps/minute)		0.92 (0.88-0.97)		0.93 (0.89-0.98)
**Baseline survival estimate at 5 years**	0.998	0.997	0.997	0.997

Data are adjusted hazard ratios with 95% confidence intervals in parenthesis. ALT = Alanine aminotransferase. AST = Aspartate aminotransferase. FP = Fractional polynomial. HDL = High-density lipoprotein.

Supplemental Table 9: Reclassification of participants after addition of accelerometer data to baseline and laboratory models for 5-year predicted mortality risk, by subgroup

	UK Biobank (N=79,717)		NHANES (N=4,713)	
	**Baseline model versus baseline plus accelerometer data**	**Laboratory model versus laboratory plus accelerometer data**	**Baseline model versus baseline plus accelerometer data**	**Laboratory model versus laboratory plus accelerometer data**
**Net reclassification index (95% CI)**				
Female	3.1% (1.8%-4.5%)	1.1% (-0.0%-2.5%)	4.1% (-0.7%-8.9%)	3.8% (-0.9%-8.4%)
Male	3.5% (1.5%-5.3%)	4.4% (2.6%-6.3%)	3.7% (-1.0%-8.4%)	-2.0% (-5.7%-1.7%)
<70 years	3.3% (2.0%-4.6%)	2.7% (1.4%-3.9%)	8.9% (2.4%-15.4%)	-0.0% (-5.5%-5.4%)
≥70 years	3.3% (1.0%-5.7%)	3.7% (1.3%-6.1%)	5.9% (1.6%-10.2%)	5.0% (1.1%-8.9%)
Prior disease	5.9% (3.1%-8.6%)	5.1% (2.5%-7.7%)	6.0% (1.6%-10.5%)	1.0% (-2.8%-4.8%)
No prior disease	1.8% (0.1%-2.8%)	2.0% (0.8%-3.2%)	1.4% (-4.2%-7.0%)	-0.3% (-5.1%-4.5%)
**Integrated discrimination improvement (95% CI)**				
Female	0.005 (0.003-0.007)	0.005 (0.003-0.007)	0.051 (0.034-0.069)	0.036 (0.022-0.051)
Male	0.007 (0.005-0.009)	0.006 (0.004-0.008)	0.049 (0.032-0.065)	0.032 (0.019-0.046)
<70 years	0.005 (0.004-0.007)	0.005 (0.003-0.007)	0.018 (0.008-0.029)	0.018 (0.008-0.027)
≥70 years	0.008 (0.005-0.011)	0.006 (0.004-0.009)	0.077 (0.059-0.095)	0.049 (0.035-0.064)
Prior disease	0.011 (0.008-0.015)	0.010 (0.006-0.013)	0.063 (0.046-0.079)	0.043 (0.030-0.057)
No prior disease	0.003 (0.002-0.004)	0.003 (0.002-0.004)	0.031 (0.016-0.047)	0.019 (0.006-0.032)

Net reclassification index based on 10% risk threshold. Baseline model: sex, age group, smoking, body mass index, systolic blood pressure, blood pressure medication, cholesterol medication, prior myocardial infarction, stroke, congestive heart failure, cancer, and diabetes. Laboratory model: baseline model plus albumin, alanine aminotransferase, aspartate aminotransferase, high density lipoprotein cholesterol, gamma glutamyltransferase, lymphocyte count, mean corpuscular haemoglobin, mean corpuscular haemoglobin concentration, red blood cell count, red blood cell distribution width, urea, and white blood cell count. Accelerometer data: daily steps and walking cadence. CI = Confidence interval. N = Number of participants. NHANES = National Health and Nutrition Examination Survey.

Supplemental Table 10: Underlying cause of death at five-years in the UK Biobank

**Underlying cause of death (ICD-10)**	**N (%)**
Vascular death (I00-I99)	334 (20.4%)
Cancer death (C00-C97)	984 (60.0%)
Other death	322 (19.6%)
Certain infectious and parasitic diseases (A)	9 (0.5%)
Diseases of the blood and blood-forming organs and certain disorders involving the immune mechanism (D)	23 (1.4%)
Endocrine, nutritional and metabolic diseases (E)	11 (0.7%)
Mental and behavioural disorders (F)	7 (0.4%)
Diseases of the nervous system (G)	52 (3.2%)
Diseases of the ear and mastoid process (H)	1 (0.1%)
Diseases of the respiratory system (J)	84 (5.1%)
Diseases of the digestive system (K)	52 (3.2%)
Diseases of the skin and subcutaneous tissue (L)	4 (0.2%)
Diseases of the musculoskeletal system and connective tissue (M)	9 (0.5%)
Diseases of the genitourinary system (N)	6 (0.4%)
Congenital malformations, deformations and chromosomal abnormalities (Q)	2 (0.1%)
Symptoms, signs and abnormal clinical and laboratory findings, not elsewhere classified (R)	6 (0.4%)
Codes for special purposes (U)	16 (1.0%)
External causes of morbidity and mortality (V-Y)	40 (2.4%)
**Any death**	**1,640**

N = Number of participants. ICD-10 = International Classification of Diseases, 10th revision.

Supplemental Table 11: Underlying cause of death at five-years in the NHANES

**Underlying cause of death (ICD-10)**	**N (%)**
Vascular death (I00-I09, I11, I13, I20-I51, and I60-I69)	114 (30.6%)
Cancer death (C00-C97)	104 (28.0%)
Other death	154 (41.4%)
Chronic lower respiratory diseases (J40-J47)	15 (4.0%)
Accidents/Unintentional injuries (V01-X59, Y85-Y86)	12 (3.2%)
Alzheimer's disease (G30)	7 (1.9%)
Diabetes mellitus (E10-E14)	16 (4.3%)
Influenza and pneumonia (J09-J18)	6 (1.6%)
Nephritis, nephrotic syndrome and nephrosis (N00-N07, N17-N19, N25-N27)	12 (3.2%)
All other causes (data unavailable)	88 (23.1%)
**Any death**	**372**

N = Number of participants. NHANES = National Health and Nutrition Examination Survey. ICD-10 = International Classification of Diseases, 10th revision.

Supplemental Table 12: 5-year mortality risk equations

Model	10-year mortality risk formula
Baseline	Linear predictor = 0.3987761 x (if male) + 0.4252677 x (if 50-54) + 0.8671005 x (if 55-59) + 1.261298 x (if 60-64) + 1.61343 x (65-69) + 2.075684 x (if 70-74) + 2.407846 x (if over 75) +0.14842 x (Body mass index (per 5 kg/m2))<br>0.81978 (if smoker) + 0.07696 (SBP/10) +<br>0.13976 (if blood pressure medication) +<br>0.03922 (if cholesterol lowering medication) +<br>0.41211 (if Myocardial infarction) +<br>0.27763 (if Stroke) +<br>0.85015 (if Congestive heart failure) +<br>1.12168 (if Cancer) +<br>0.41871 (if Diabetes)<br><br>10-year mortality risk score = $0.988^{e^{\text{Linear predictor}}}$
Baseline model<br>plus accelerometer data	Linear predictor =<br>0.42527 X (if male) +<br>0.41871X (if 50-54) +<br>0.85866 X (if 55-59) +<br>1.23547 X (if 60-64) +<br>1.57070 X (65-69) +<br>1.98513 X (if 70-74) +<br>2.24918 X (if over 75) +<br>0.69315 X (if smoker) +<br>0.04879 X (Body mass index (per 5 kg/m2)<br>0.07696 X (SBP/10) +<br>0.09531 X (if blood pressure medication) +<br>0.03922 X (if cholesterol lowering medication) +<br>0.37156 X (if Myocardial infarction) +<br>0.14842 X (if Stroke) +<br>0.68813 X (if Congestive heart failure) +<br>1.08181 X (if Cancer) +<br>0.32208 X (if Diabetes) +<br><br>-0.44629 X (if Daily steps/1000) +<br>0.12222 X (if Daily steps/1000) +<br>-0.08338 X (if Walking cadence per 10 steps/minute) +<br><br>10-year mortality risk score = $0.997^{e^{\text{Linear predictor}}}$
Laboratory model	Linear predictor =<br>0.35767 X (if male) +<br>0.41211X (if 50-54) +<br>0.83725 X (if 55-59) +<br>1.24127 X (if 60-64) +<br>1.58515X(65-69) +<br>2.00013 X (if 70-74) +<br>2.28544 X (if over 75) +<br>0.63658 X (if smoker) +<br>0.12222 X (Body mass index (per 5 kg/m2)<br>0.08618 (SBP/10) +<br>0.06766 X (if blood pressure medication) +<br>0.04879 X (if cholesterol lowering medication) +<br>0.35066 X (if Myocardial infarction) +

	0.19062 X (if Stroke) +<br>0.73716 X (if Congestive heart failure) +<br>1.06815 X (if Cancer) +<br>0.36464 X (if Diabetes) +<br><br>-0.43078 X (Albumin (per 10 g/L))<br>- 0.08338 X (ALT (per 10 U/L))<br>0.06766 X (AST (per 10 U/L) FP1)<br>- 0.02020X (AST (per 10 U/L) FP2)<br>- 0.12783 X (HDL cholesterol (per mmol/L))<br>0.13103 X (Gamma glutamyltransferase (per 100 U/L))<br>0.47000 X (Lymphocyte count (per 10^9 cells/Litre))<br>0.85015 X (Mean corpuscular haemoglobin (per 10 pg))<br>- 0.03046X (Mean corpuscular haemoglobin concentration (per 10 g/dL)<br>- 0.04082 X (Red blood count (per 10^12 cells/L) FP1)<br>0.01980 X (Red blood count (per 10^12 cells/L) FP2)<br>1.32972 X (Red blood distribution width (per 10%))<br>1.49962 X (Urea (per 100 mmol/L) FP1)<br>3.13810 X (Urea (per 100 mmol/L) FP2)<br>0.90826 X (White blood count (per 10^10 cells/L) FP1)<br>-0.26136 X (White blood count (per 10^10 cells/L) FP2)<br><br>10-year mortality risk score = $0.997^{e^{\text{Linear predictor}}}$
Laboratory model plus accelerometer data	Linear predictor =<br>0.39878 X (if male) +<br>0.41211 X (if 50-54) +<br>0.82418 X (if 55-59) +<br>1.20597 X (if 60-64) +<br>1.52823 X (65-69) +<br>1.90211 X (if 70-74) +<br>2.12942 X (if over 75) +<br>0.53649 X (if smoker) +<br>0.03922 X (Body mass index (per 5 kg/m2)<br>0.08618 X (SBP/10) +<br>0.03922 X (if blood pressure medication) +<br>0.04879 X (if cholesterol lowering medication) +<br>0.32208 X (if Myocardial infarction) +<br>0.08618 X (if Stroke) +<br>0.61519 X (if Congestive heart failure) +<br>1.04028 X (if Cancer) +<br>0.29267 X (if Diabetes) +<br><br>- 0.40048X (Albumin (per 10 g/L))+<br>- 0.08338X (ALT (per 10 U/L))+<br>0.06766X (AST (per 10 U/L) FP1)+<br>- 0.02020X (AST (per 10 U/L) FP2)+<br>- 0.07257X (HDL cholesterol (per mmol/L))+<br>0.09531X (Gamma glutamyltransferase (per 100 U/L))+<br>0.38526X (Lymphocyte count (per 10^9 cells/Litre)) +<br>0.79299 X (Mean corpuscular haemoglobin (per 10 pg)) +<br>- 0.03046 X (Mean corpuscular haemoglobin concentration (per 10 g/dL)<br>- 0.04082 X (Red blood count (per 10^12 cells/L) FP1) +<br>0.01980 X (Red blood count (per 10^12 cells/L) FP2) +<br>1.19089 X (Red blood distribution width (per 10%)) +

	1.24415 X (Urea (per 100 mmol/L) FP1)+
	2.63261 X (Urea (per 100 mmol/L) FP2)+
	0.74669 X (White blood count (per 10^10 cells/L) FP1) +
	- 0.21072 X (White blood count (per 10^10 cells/L) FP2) +
	-0.38566 X (if Daily steps/1000) +
	0.10436 X (if Daily steps/1000) +
	- 0.07257X (if Walking cadence per 10 steps/minute) +
	10-year mortality risk score = $0.997^{e^{\text{Linear predictor}}}$

Supplemental Table 13: Example 5-year mortality risk score of 65-year-old male, smoker, 27 BMI,160 mm Hg SBP, no blood pressure medication, Cholesterol lowering medication, prior diabetes, 7000 steps per day using the full Laboratory model plus accelerometer model

Risk factors	Original input value	Predictor value after required transformation	Coefficient	Product of predictor value and coefficient
Male	True	1	0.39878	0.39878
Age group				
<50 years	-	0	0.41211	0
50-54 years	-	0	0.82418	0
55-59 years	-	0	1.20597	0
60-64 years	-	0	1.52823	0
65-69 years	True	1	1.90211	1.90211
70-74 years	-	0	2.12942	0
≥75 years	-	0	0.53649	0
Current smoker	True	1	0.53649	0.53649
Body mass index (per 5 kg/m^2)	27	27/10 – 29.5/10 = -0.25	0.03922	-0.009805
Systolic blood pressure mmHg	160	160/10 – 128/10 = 3.2	0.08618	0.275776
Blood pressure medication	False	0	0.03922	0
Cholesterol lowering medication	True	1	0.04879	0.04879
**Prior disease**				
Myocardial infarction	False	0	0.32208	0
Stroke	False	0	0.08618	0
Congestive heart failure	False	0	0.61519	0
Cancer	False	0	1.04028	0
Diabetes	True	1	0.29267	0.29267
**Laboratory measures**				
Albumin (per 10 g/L)	44.1	44.1/10 – 42.2/10 = 0.19000000	-0.40048	-0.076091200
ALT (per 10 U/L)	20.0	20.0/10 – 24.3/10 = -0.43000000	- 0.08338	0.035853400
AST (per 10 U/L) FP1	30	$(30/10)^2$ - 7.541961 = 1.45803900	0.06766	0.098650919
AST (per 10 U/L) FP2	30	(log (30/10) X $(30/10)^2$) – 8.886379 = 1.001132	-0.02020	-0.020222866
HDL cholesterol (per mmol/L)	1.2	1.2 – 1.4 = -0.2	-0.07257	0.014514000
Gamma glutamyltransferase (per 100 U/L)	32.3	32.3/100 – 0.3415259 = -0.01852590	0.09531	-0.001765704
Lymphocyte count (per 10^9 cells/Litre)	2.6	$2.6^{-0.5}$ - 0.7499859 = -0.1298122	0.38526	-0.050011448
Mean corpuscular haemoglobin (per 10 pg)	29.2	29.2/10 – 33.9/10 = -0.47	0.79299	-0.372705300
Mean corpuscular haemoglobin concentration (per 10 g/dL)	34.1	$(34.1/10)^3$ –41.23892= -1.587099	- 0.03046	0.048343036
Red blood count (per 10^12 cells/L) FP1	4.8	4.8^3 – 93.69396 = 16.89804	-0.04082	-0.689777993

Red blood count (per 10^12 cells/L) FP2	4.8	log (4.8) X 4.8^3 - 142.9003 = 30.57607	0.01980	0.605406186
Red blood distribution width (per 10%)	12.5	12.5/10 -13.5/10 = -0.1	1.19089	-0.119089000
Urea (per 100 mmol/L) FP1	5.3	$(5.3/100)^{-0.5}$ - 4.41876 = -0.07503757	1.24415	-0.093357993
Urea (per 100 mmol/L) FP2	5.3	log (5.3/100) – -2.957519 = 0.02005563	2.63261	0.052798652
White blood count (per 10^10 cells/L) FP1	6.5	$(6.5/10)^2$ - 0.4809559 = -0.0584559	0.74669	-0.043648436
White blood count (per 10^10 cells/L) FP2	6.5	$(6.5/10)^3$ - 0.4084449 = -0.1338199	-0.21072	0.028198529
**Accelerometer data**				
Daily steps (per 1000 steps) FP1	7000	7000/1000 – 9.462049 = -2.462049	-0.38566	0.949513817
Daily steps (per 1000 steps) FP2	7000	7000/1000 * log (7000/1000) – 22.03024 = -8.408869	0.10436	-0.877549569
Walking cadence (per 10 steps/minute)	90 - 103	90/10 – 103/10 = -1.3	- 0.07257	0.094341000
$5 - year\ risk = 100 * 1 - 0.997^{e^{3.028211}}$ = 6.02 %				

Supplemental Figure 1: Flow chart for UK Biobank participants included in analyses

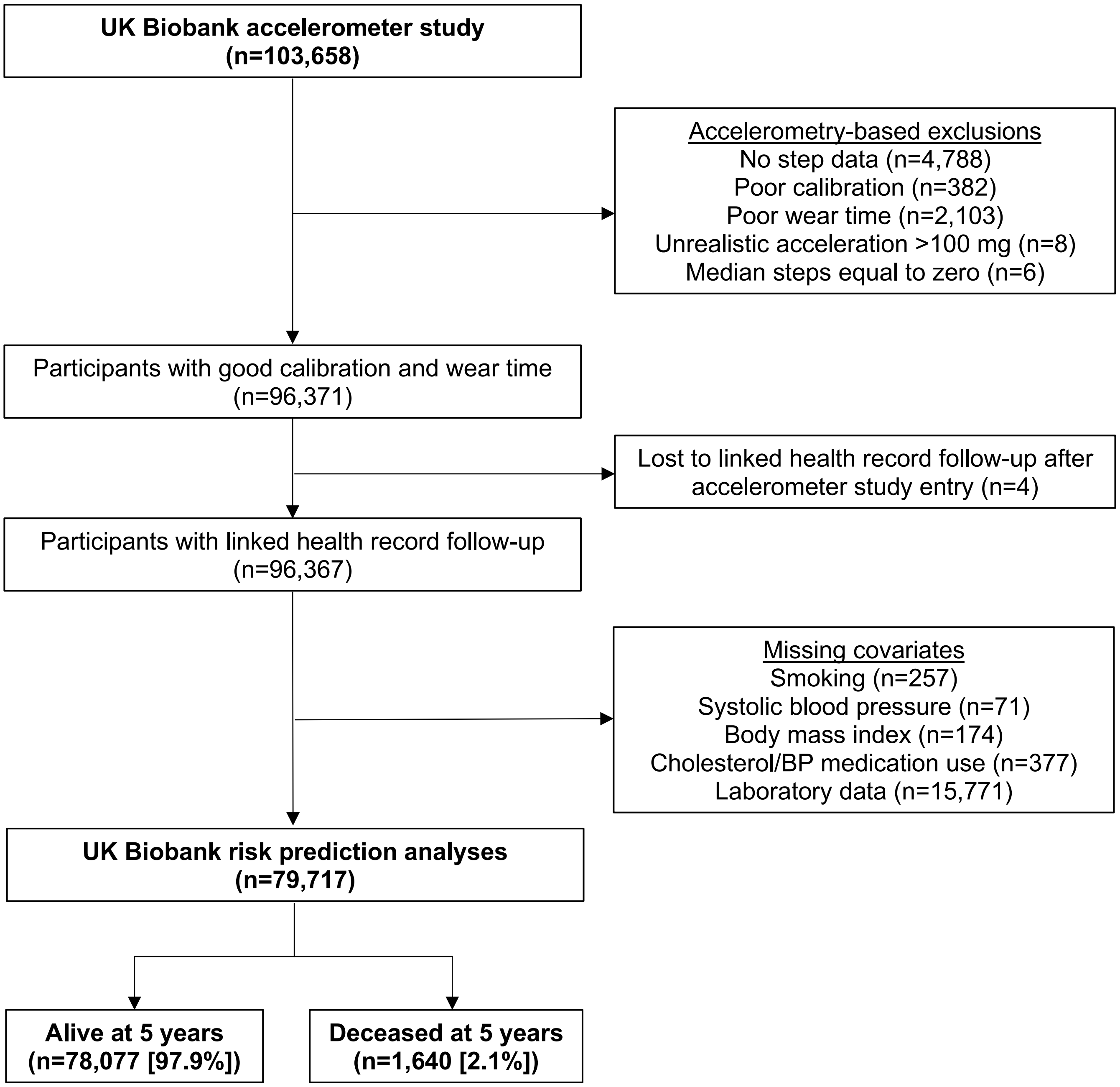

BP = Blood pressure. N = Number of participants.

Supplemental Figure 2: Flow chart for NHANES participants included in analyses

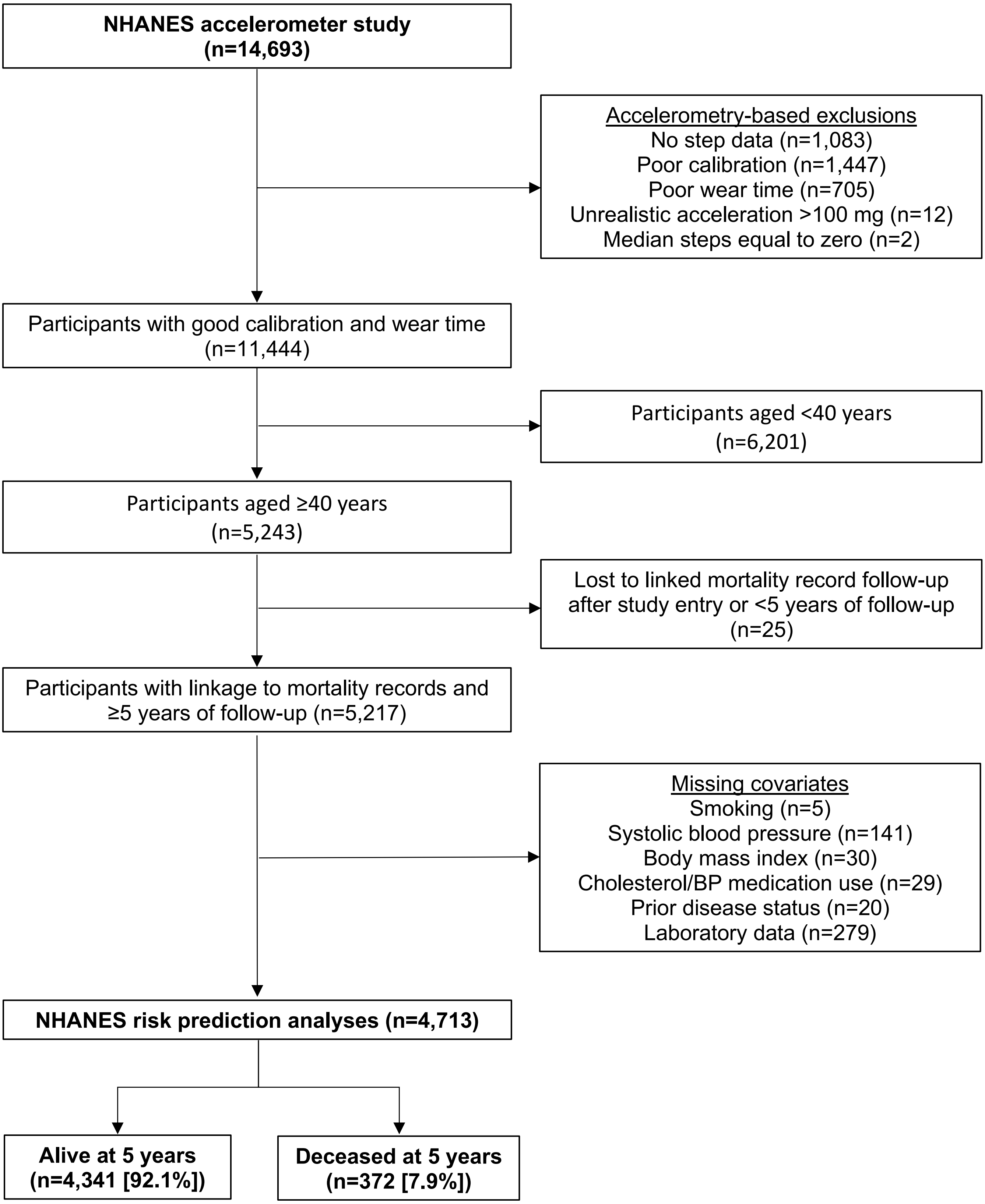

BP = Blood pressure. N = Number of participants. NHANES = National Health and Nutrition Examination Survey.

Supplemental Figure 3: Calibration of laboratory models for 5-year all-cause mortality risk in the UK Biobank and the NHANES, by predicted risk decile

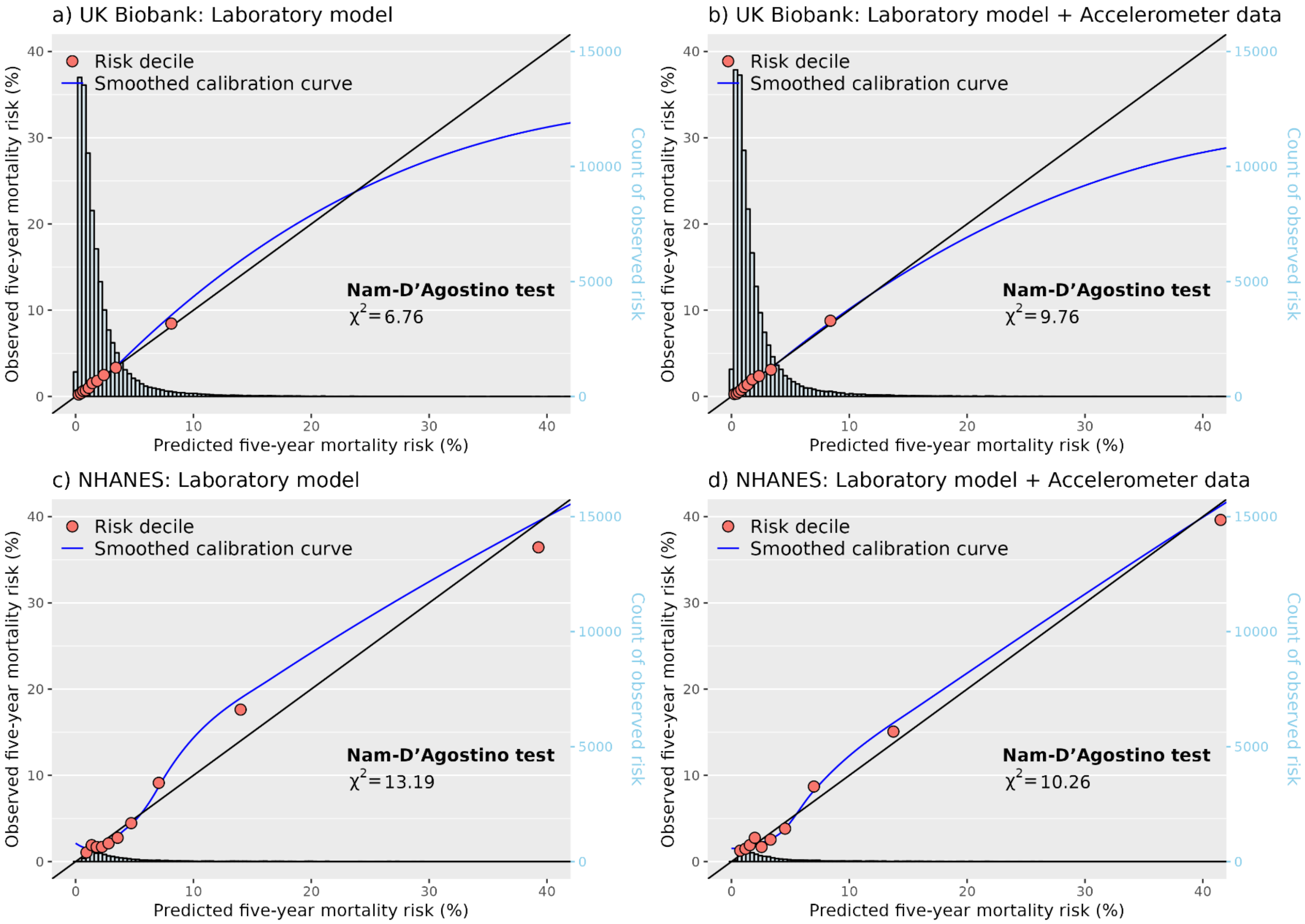

Red circles represent the observed five-year mortality risk at each decile of predicted five-year risk. Black line represents perfect calibration. Blue line represents the smoothed calibration curve of all risk deciles generated using jack-knife methods. Data for calibration plots generated using five-fold cross validation. Calibration performance estimated using Nam-D'Agostino test with nine degrees of freedom, where the smaller values represents greater performance. NHANES = National Health and Nutrition Examination Survey.

Supplemental Figure 4: Discrimination improvement after addition of accelerometer data to baseline model for 5-year predicted mortality risk in the UK Biobank, by subgroup

	N	Deaths	C-index (95% CI) Baseline model	C-index (95% CI) Baseline model plus accelerometer data	Change in C-index (95% CI)
**Sex**					
Female	44,410	640	0.736 (0.717, 0.756)	0.746 (0.727, 0.765)	+0.010 (+0.004, +0.016)
Male	35,307	1,000	0.758 (0.744, 0.772)	0.768 (0.754, 0.782)	+0.010 (+0.005, +0.015)
**Age**					
<70 years	65,021	960	0.747 (0.732, 0.763)	0.755 (0.740, 0.770)	+0.007 (+0.003, +0.012)
70+ years	14,696	680	0.668 (0.646, 0.690)	0.684 (0.662, 0.705)	+0.016 (+0.007, +0.026)
**Prior disease**					
Yes	10,856	640	0.668 (0.648, 0.689)	0.696 (0.676, 0.716)	+0.028 (+0.019, +0.039)
No	68,861	1,000	0.728 (0.712, 0.743)	0.735 (0.719, 0.750)	+0.007 (+0.002, +0.012)
**Overall**	**79,717**	**1,640**	**0.762 (0.752, 0.774)**	**0.770 (0.760, 0.782)**	**+0.008 (+0.005, +0.011)**

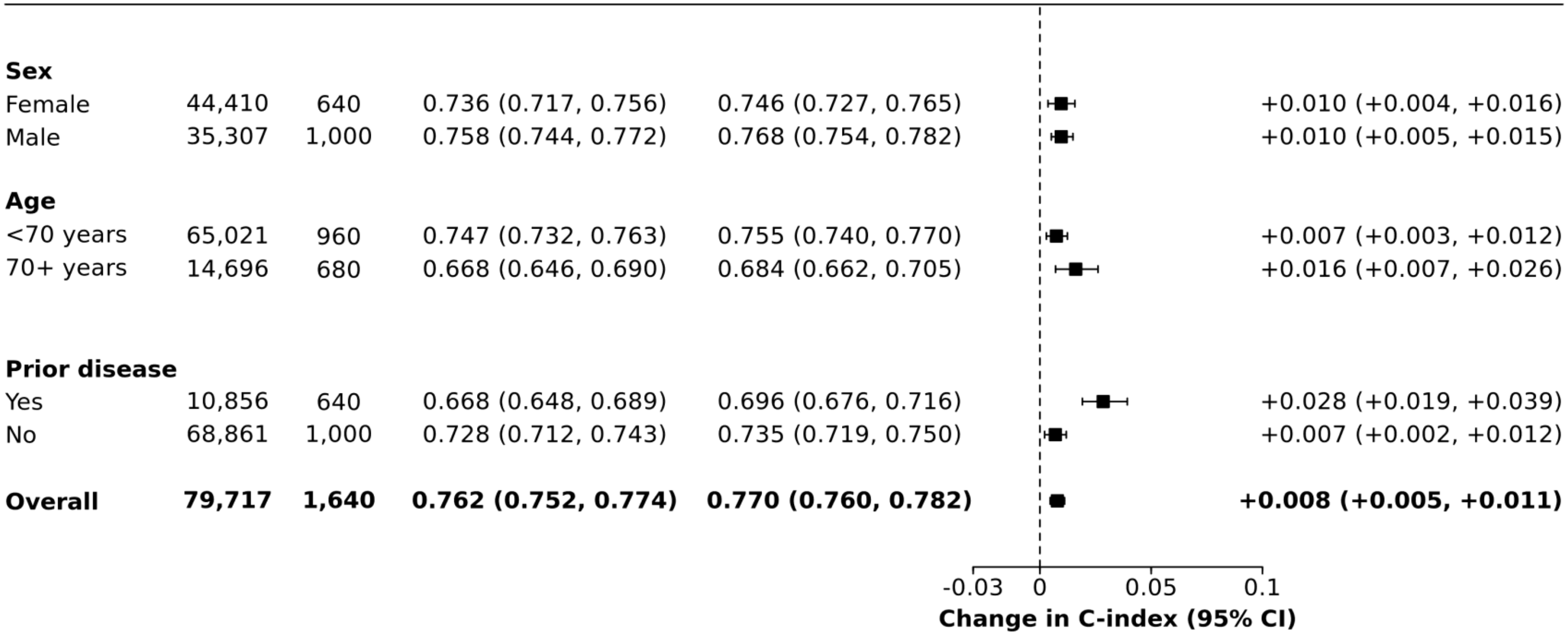

Squares represent the change in c-index compared to the baseline model and horizontal lines are 95% confidence intervals generated using 1,000 bootstrap samples. Baseline model: sex, age group, smoking, body mass index, systolic blood pressure, blood pressure medication, cholesterol medication, prior myocardial infarction, stroke, congestive heart failure, cancer, and diabetes. Accelerometer data: steps and walking cadence. Prior disease includes myocardial infarction, stroke, congestive heart failure, cancer, and diabetes. CI = Confidence interval. N = Number of participants.

Supplemental Figure 5: Discrimination improvement after addition of accelerometer data to laboratory model for 5-year predicted mortality risk in the UK Biobank, by subgroup

	N	Deaths	C-index (95% CI) Laboratory model	C-index (95% CI) Laboratory and accelerometer data	Change in C-index (95% CI)
**Sex**					
Female	44,410	640	0.745 (0.725, 0.764)	0.751 (0.732, 0.770)	+0.007 (+0.002, +0.012)
Male	35,307	1,000	0.772 (0.759, 0.787)	0.778 (0.764, 0.793)	+0.005 (+0.002, +0.009)
**Age**					
<70 years	65,021	960	0.760 (0.745, 0.776)	0.765 (0.749, 0.781)	+0.005 (+0.001, +0.009)
70+ years	14,696	680	0.685 (0.664, 0.706)	0.695 (0.673, 0.716)	+0.010 (+0.003, +0.017)
**Prior disease**					
Yes	10,856	640	0.685 (0.665, 0.706)	0.705 (0.686, 0.726)	+0.020 (+0.013, +0.029)
No	68,861	1,000	0.742 (0.726, 0.756)	0.745 (0.730, 0.760)	+0.004 (0.000, +0.008)
**Overall**	**79,717**	**1,640**	**0.773 (0.762, 0.785)**	**0.778 (0.767, 0.789)**	**+0.005 (+0.002, +0.007)**

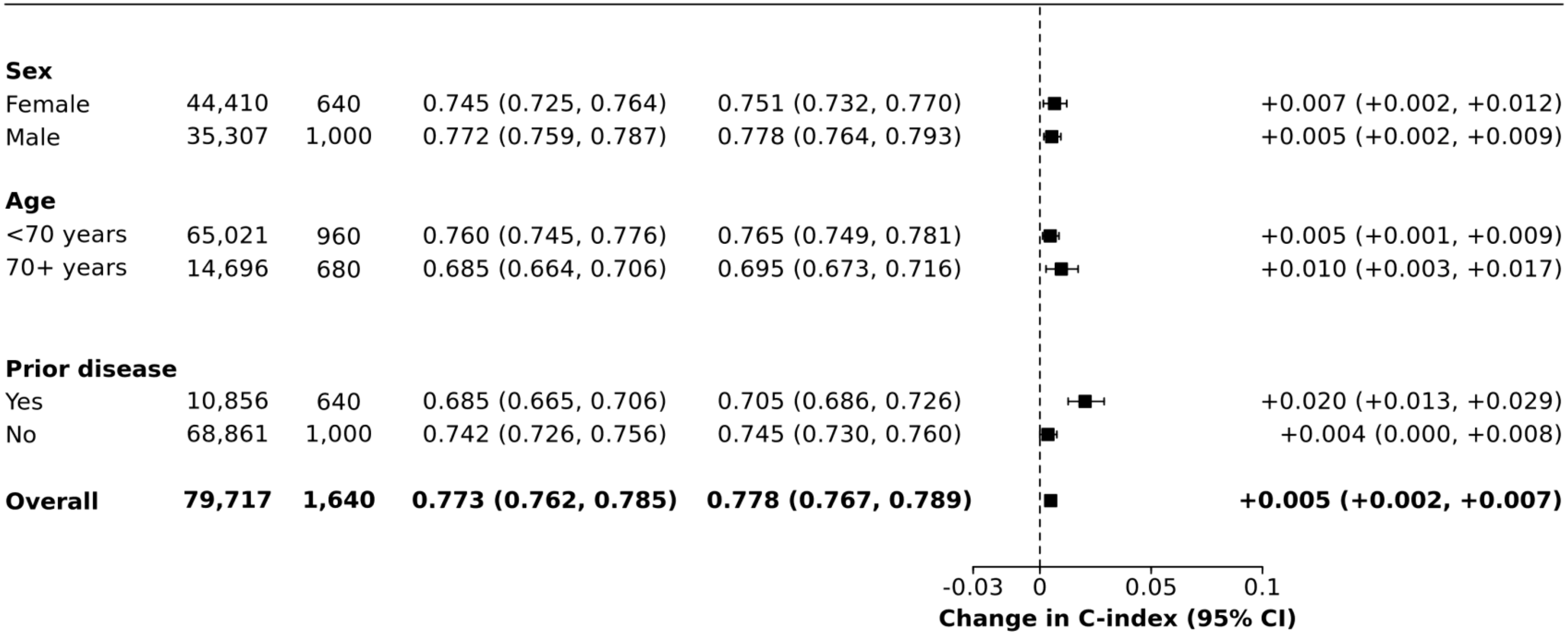

Squares represent the change in c-index compared to the laboratory model and horizontal lines are 95% confidence intervals generated using 1,000 bootstrap samples for each subgroup. Baseline model: sex, age group, smoking, body mass index, systolic blood pressure, blood pressure medication, cholesterol medication, prior myocardial infarction, stroke, congestive heart failure, cancer, and diabetes. Laboratory model: baseline model plus albumin, alanine aminotransferase, aspartate aminotransferase, high density lipoprotein cholesterol, gamma glutamyltransferase, lymphocyte count, mean corpuscular haemoglobin, mean corpuscular haemoglobin concentration, red blood cell count, red blood cell distribution width, urea, and white blood cell count. Accelerometer data: steps and walking cadence. Prior disease includes myocardial infarction, stroke, congestive heart failure, cancer, and diabetes. CI = Confidence interval. N = Number of participants.

Supplemental Figure 6: Discrimination improvement after addition of accelerometer data to baseline model for 5-year predicted mortality risk in the NHANES, by subgroup

	N	Deaths	C-index (95% CI) Baseline model	C-index (95% CI) Baseline model plus accelerometer data	Change in C-index (95% CI)
**Sex**					
Female	2,432	166	0.801 (0.766, 0.833)	0.820 (0.788, 0.851)	+0.019 (+0.005, +0.036)
Male	2,281	206	0.797 (0.767, 0.824)	0.808 (0.781, 0.837)	+0.012 (-0.002, +0.027)
**Age**					
<70 years	3,593	130	0.702 (0.659, 0.747)	0.716 (0.672, 0.762)	+0.013 (-0.012, +0.042)
70+ years	1,120	242	0.697 (0.665, 0.731)	0.753 (0.720, 0.782)	+0.056 (+0.034, +0.080)
**Prior disease**					
Yes	1,528	235	0.741 (0.708, 0.772)	0.778 (0.746, 0.805)	+0.036 (+0.020, +0.053)
No	3,185	137	0.778 (0.733, 0.818)	0.785 (0.743, 0.824)	+0.007 (-0.013, +0.031)
**Overall**	**4,713**	**372**	**0.801 (0.778, 0.820)**	**0.816 (0.795, 0.835)**	**+0.015 (+0.007, +0.025)**

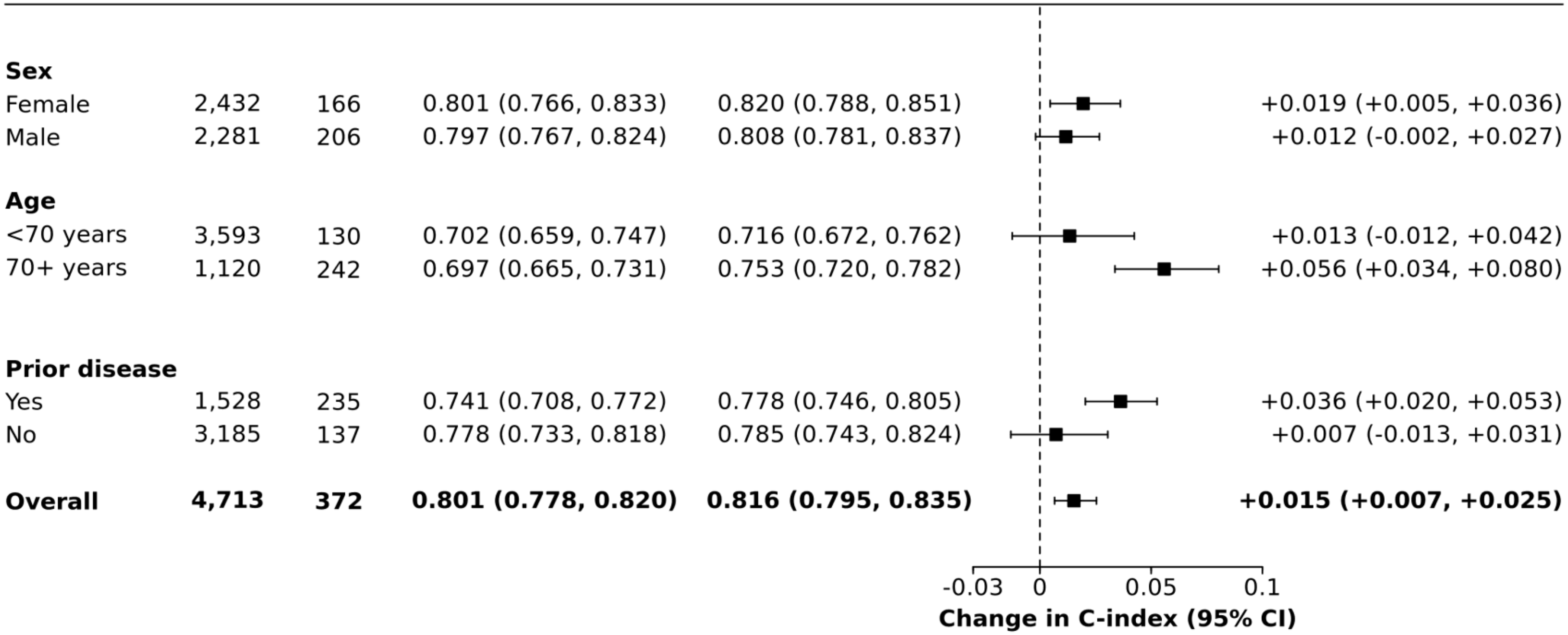

Squares represent the change in c-index compared to the baseline model and horizontal lines are 95% confidence intervals generated using 1,000 bootstrap samples. Baseline model: sex, age group, smoking, body mass index, systolic blood pressure, blood pressure medication, cholesterol medication, prior myocardial infarction, stroke, congestive heart failure, cancer, and diabetes. Accelerometer data: steps and walking cadence. Prior disease includes myocardial infarction, stroke, congestive heart failure, cancer, and diabetes. CI = Confidence interval. N = Number of participants. NHANES = National Health and Nutrition Examination Survey.

Supplemental Figure 7: Discrimination improvement after addition of accelerometer data to laboratory model for 5-year predicted mortality risk in the NHANES, by subgroup

	N	Deaths	C-index (95% CI): Laboratory model	C-index (95% CI): Laboratory model plus accelerometer data	Change in C-index (95% CI)
**Sex**					
Female	2,432	166	0.836 (0.808, 0.864)	0.843 (0.815, 0.871)	+0.007 (-0.003, +0.017)
Male	2,281	206	0.825 (0.799, 0.852)	0.827 (0.801, 0.854)	+0.002 (-0.007, +0.011)
**Age**					
<70 years	3,593	130	0.768 (0.727, 0.810)	0.756 (0.713, 0.801)	-0.012 (-0.030, +0.006)
70+ years	1,120	242	0.736 (0.702, 0.767)	0.770 (0.740, 0.798)	+0.034 (+0.019, +0.053)
**Prior disease**					
Yes	1,528	235	0.786 (0.756, 0.813)	0.803 (0.774, 0.830)	+0.018 (+0.008, +0.028)
No	3,185	137	0.812 (0.775, 0.845)	0.809 (0.774, 0.843)	-0.003 (-0.017, +0.013)
**Overall**	**4,713**	**372**	**0.831 (0.812, 0.850)**	**0.836 (0.816, 0.856)**	**+0.005 (-0.001, +0.011)**

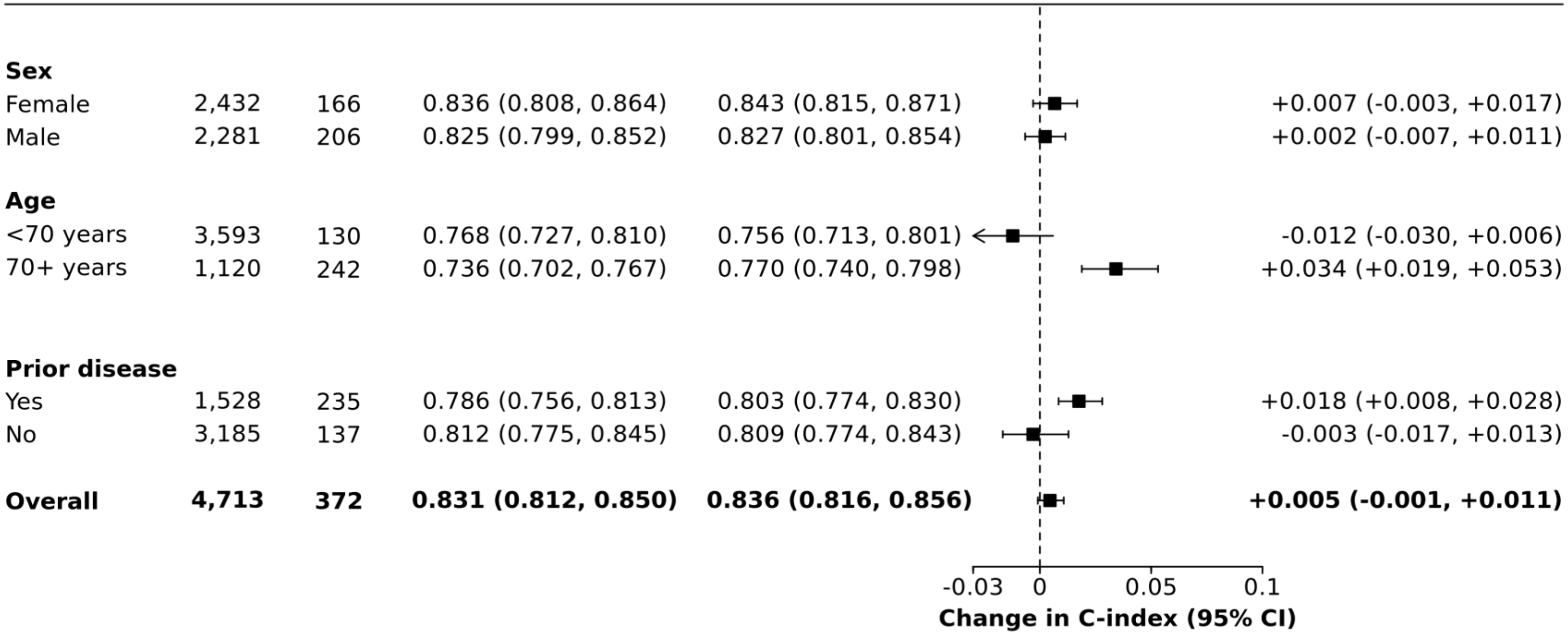

Squares represent the change in c-index compared to the laboratory model and horizontal lines are 95% confidence intervals generated using 1000 bootstrap samples for each subgroup. Baseline model: sex, age group, smoking, body mass index, systolic blood pressure, blood pressure medication, cholesterol medication, prior myocardial infarction, stroke, congestive heart failure, cancer, and diabetes. Laboratory model: baseline model plus albumin, alanine aminotransferase, aspartate aminotransferase, high density lipoprotein cholesterol, gamma glutamyltransferase, lymphocyte count, mean corpuscular haemoglobin, mean corpuscular haemoglobin concentration, red blood cell count, red blood cell distribution width, urea, and white blood cell count. Accelerometer data: steps and walking cadence. Prior disease includes myocardial infarction, stroke, congestive heart failure, cancer, and diabetes. CI = Confidence interval. N = Number of participants. National Health and Nutrition Examination Survey.

Supplemental Figure 8: Discrimination improvement after addition of accelerometer data to baseline model for 5-year predicted mortality risk, by cause of death

	Deaths	C-index (95% CI)		Change in C-index (95% CI)
		Baseline model	Baseline model plus accelerometer data	
**UK Biobank**				
Vascular death	334	0.795 (0.773, 0.818)	0.798 (0.776, 0.820)	+0.003 (-0.005, +0.011)
Cancer death	984	0.747 (0.732, 0.762)	0.751 (0.737, 0.767)	+0.004 (+0.001, +0.009)
Other death	322	0.775 (0.750, 0.801)	0.799 (0.773, 0.824)	+0.023 (+0.015, +0.034)
**All-cause mortality**	**1,640**	**0.762 (0.752, 0.774)**	**0.770 (0.760, 0.782)**	**+0.008 (+0.005, +0.011)**
**NHANES**				
Vascular death	114	0.854 (0.824, 0.886)	0.864 (0.834, 0.898)	+0.010 (-0.005, +0.027)
Cancer death	104	0.770 (0.725, 0.812)	0.767 (0.716, 0.808)	-0.003 (-0.026, +0.019)
Other death	154	0.782 (0.744, 0.818)	0.813 (0.778, 0.847)	+0.031 (+0.015, +0.051)
**All-cause mortality**	**372**	**0.801 (0.778, 0.820)**	**0.816 (0.795, 0.835)**	**+0.015 (+0.007, +0.025)**

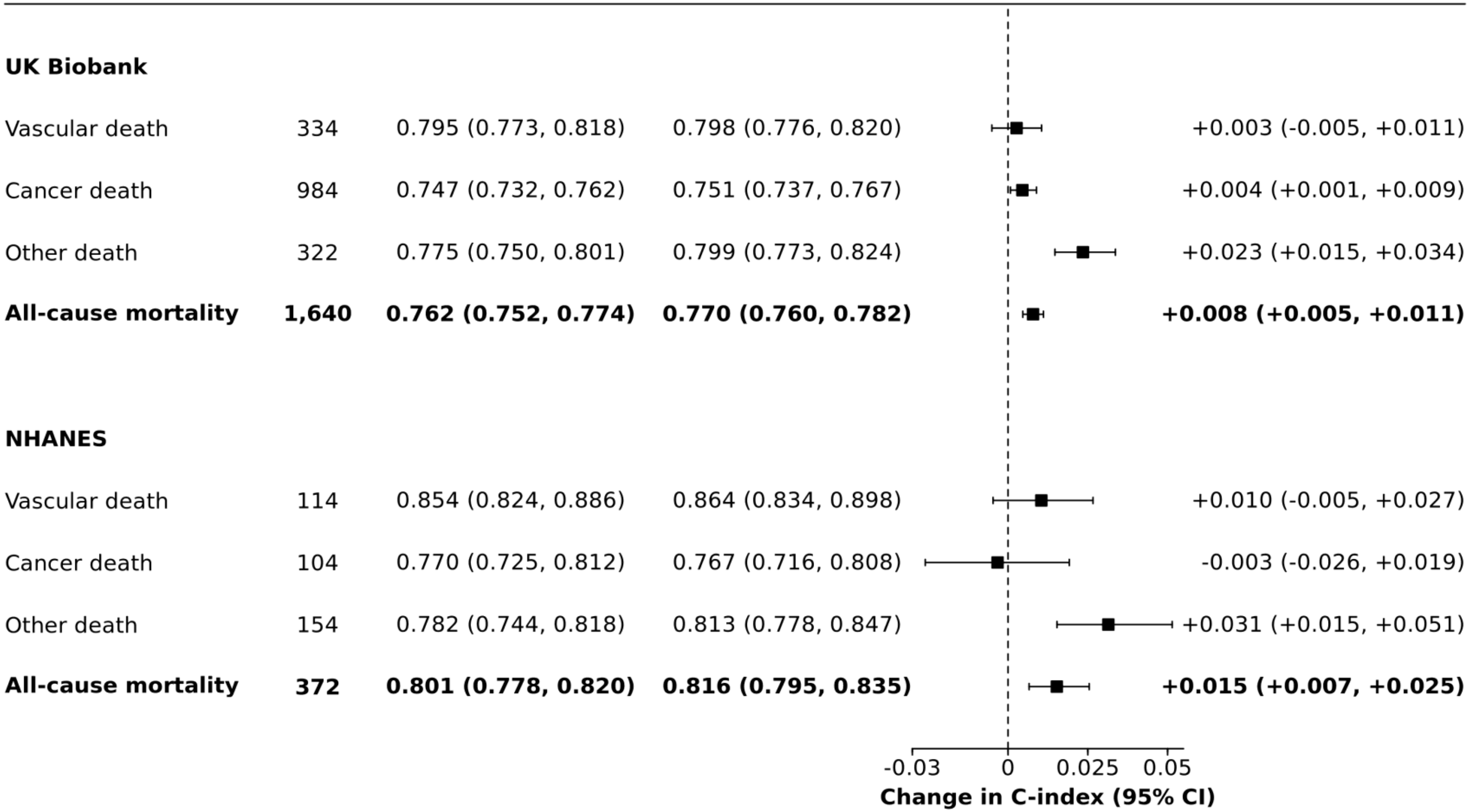

Squares represent the change in c-index comparted to the baseline model and horizontal lines are 95% confidence intervals generated using 1,000 bootstrap samples. Baseline model: sex, age group, smoking, body mass index, systolic blood pressure, blood pressure medication, cholesterol medication, prior myocardial infarction, stroke, congestive heart failure, cancer, and diabetes. Accelerometer data: daily steps, and walking cadence. See **Supplemental Tables 10 and 11** for cause of death definitions. CI = Confidence interval. N = Number of participants. NHANES = National Health and Nutrition Examination Survey.

Supplemental Figure 9: Discrimination improvement after addition of accelerometer data to laboratory model for 5-year predicted mortality risk, by cause of death

	Deaths	C-index (95% CI): Laboratory model	C-index (95% CI): Laboratory model plus accelerometer data	Change in C-index (95% CI)
**UK Biobank**				
Vascular death	334	0.812 (0.790, 0.831)	0.811 (0.790, 0.830)	-0.001 (-0.008, +0.006)
Cancer death	984	0.750 (0.735, 0.766)	0.754 (0.739, 0.769)	+0.004 (0.000, +0.008)
Other death	322	0.802 (0.776, 0.829)	0.816 (0.790, 0.841)	+0.014 (+0.008, +0.021)
**All-cause mortality**	**1,640**	**0.773 (0.762, 0.785)**	**0.778 (0.767, 0.789)**	**+0.005 (+0.002, +0.007)**
**NHANES**				
Vascular death	114	0.857 (0.824, 0.893)	0.866 (0.831, 0.901)	+0.008 (-0.002, +0.020)
Cancer death	104	0.786 (0.745, 0.825)	0.781 (0.737, 0.820)	-0.005 (-0.020, +0.011)
Other death	154	0.842 (0.811, 0.870)	0.850 (0.819, 0.879)	+0.008 (-0.002, +0.019)
**All-cause mortality**	**372**	**0.831 (0.812, 0.850)**	**0.836 (0.816, 0.856)**	**+0.005 (-0.001, +0.011)**

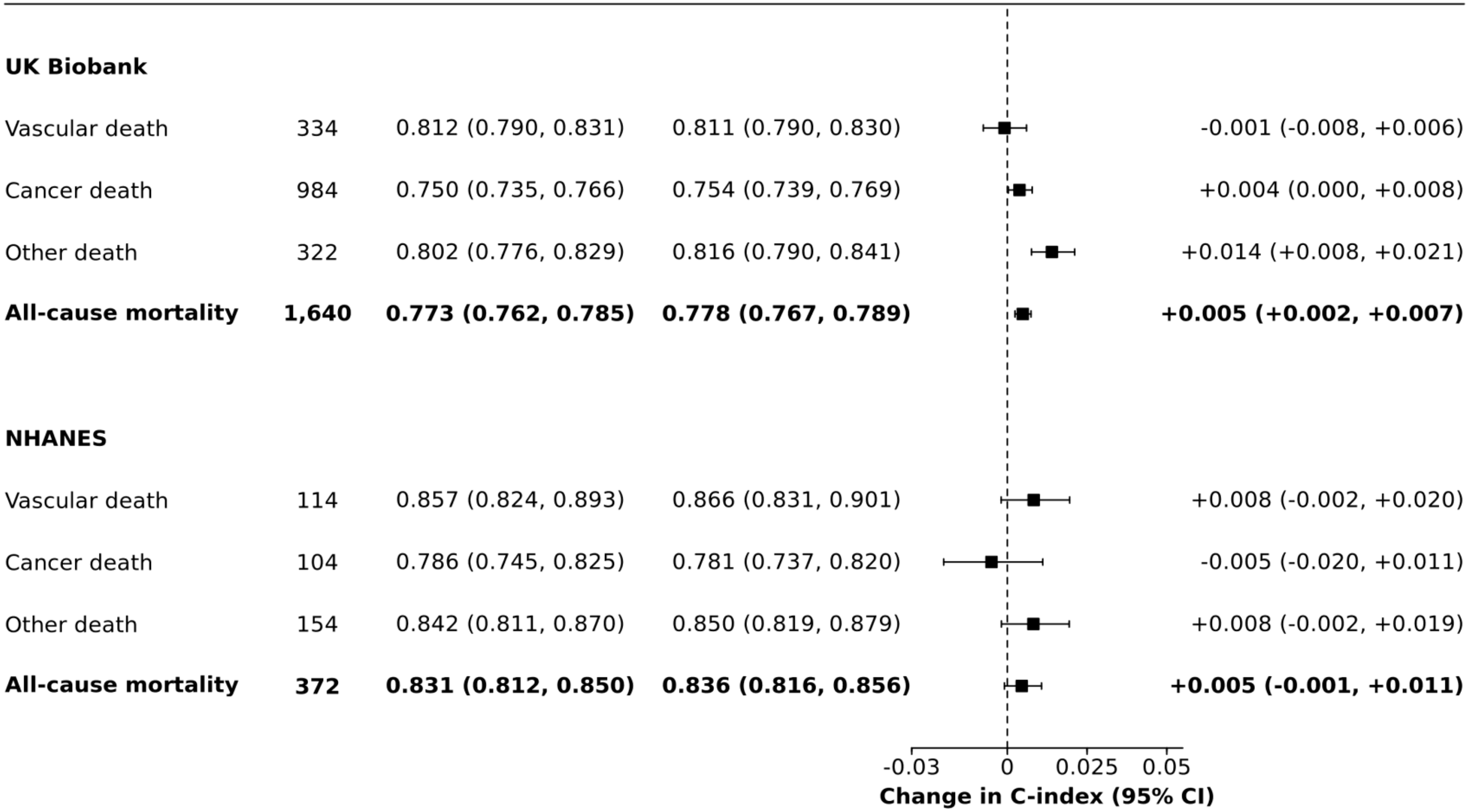

Squares represent the change in c-index comparted to the laboratory model and horizontal lines are 95% confidence intervals generated using 1,000 bootstrap samples. Baseline model: sex, age group, smoking, body mass index, systolic blood pressure, blood pressure medication, cholesterol medication, prior myocardial infarction, stroke, congestive heart failure, cancer, and diabetes. Laboratory model: baseline model plus albumin, alanine aminotransferase, aspartate aminotransferase, high density lipoprotein cholesterol, gamma glutamyltransferase, lymphocyte count, mean corpuscular haemoglobin, mean corpuscular haemoglobin concentration, red blood cell count, red blood cell distribution width, urea, and white blood cell count. Accelerometer data: daily steps, and walking cadence. See **Supplemental Tables 10 and 11** for cause of death definitions. CI = Confidence interval. N = Number of participants. NHANES = National Health and Nutrition Examination Survey.
